# RB Regulates DNA Double Strand Break Repair Pathway Choice by Mediating CtIP Dependent End Resection

**DOI:** 10.3390/ijms21239176

**Published:** 2020-12-01

**Authors:** Yuning Jiang, Jason C. Yam, Clement C. Tham, Chi Pui Pang, Wai Kit Chu

**Affiliations:** Department of Ophthalmology & Visual Sciences, The Chinese University of Hong Kong, Hong Kong, China; yuning.j@link.cuhk.edu.hk (Y.J.); yamcheuksing@cuhk.edu.hk (J.C.Y.); clemtham@cuhk.edu.hk (C.C.T.); cppang@cuhk.edu.hk (C.P.P.)

**Keywords:** RB, CtIP, homologous recombination, classical non-homologous end joining, micro-homology mediated end joining, resection

## Abstract

Inactivation of the retinoblastoma tumor suppressor gene (*RB1*) leads to genome instability, and can be detected in retinoblastoma and other cancers. One damaging effect is causing DNA double strand breaks (DSB), which, however, can be repaired by homologous recombination (HR), classical non-homologous end joining (C-NHEJ), and micro-homology mediated end joining (MMEJ). We aimed to study the mechanistic roles of RB in regulating multiple DSB repair pathways. Here we show that HR and C-NHEJ are decreased, but MMEJ is elevated in RB-depleted cells. After inducing DSB by camptothecin, RB co-localizes with CtIP, which regulates DSB end resection. RB depletion leads to less RPA and native BrdU foci, which implies less end resection. In RB-depleted cells, less CtIP foci, and a lack of phosphorylation on CtIP Thr847, are observed. According to the synthetic lethality principle, based on the altered DSB repair pathway choice, after inducing DSBs by camptothecin, RB depleted cells are more sensitive to co-treatment with camptothecin and MMEJ blocker poly-ADP ribose polymerase 1 (PARP1) inhibitor. We propose a model whereby RB can regulate DSB repair pathway choice by mediating the CtIP dependent DNA end resection. The use of PARP1 inhibitor could potentially improve treatment outcomes for RB-deficient cancers.

## 1. Introduction

Retinoblastoma tumor suppressor gene (*RB1*) has been recognized as a tumor suppressor for its roles in controlling gene transcription, cell cycle progression, and apoptosis [1,2]. Additional roles of RB in human cells have been reported, including regulating cell differentiation, preventing mitochondrial defects, and altering glucose and glutamine metabolism and apoptosis [3,4,5,6,7]. Inactivation of RB leads to genomic instability [8]. These studies suggested that RB loss could lead to a failure to maintain chromosome integrity, which is related to inappropriate DNA double strand breaks (DSB) repair, oncogenic transformation, and chromosomal translocations [9]. DSBs disrupt genomic integrity, which can be repaired by various pathways. In addition, inappropriate DSB repair can also lead to genome instability, which is related to tumor initiation, cancer progression, and possibly, cancer therapy resistance [9,10]. The repair of DSBs involves several possible mechanisms. Typically, cells employ two major pathways to repair DSBs, namely homologous recombination (HR) and classical non-homologous end joining (C-NHEJ). C-NHEJ rejoins DSBs with little or no sequence homology through a protein complex comprising Ku70/80, DNA-PKcs, and DNA ligase IV. This mechanism operates throughout the cell cycles, with its activity increasing from the G1 to G2/M phases [11]. It has been reported that RB forms protein complexes with XRCC5 and XRCC6 to support C-NHEJ [9]. HR mainly repairs DSBs in the S and G2 phases of the cell cycle [12]. In these two cell cycle stages, DNA is replicated or has been replicated. Thus, the sister chromatid carries identical DNA sequences to serve as a template for HR [13]. During HR, an important early step involves a directional 5′ to 3′ resection of DSBs to generate a 3′ single-strand DNA (ssDNA) tail [14]. End resection has been proposed to be a major determinant of the pathway choices between HR and C-NHEJ [15]. Previous studies identified multiple proteins involved in this end resection reaction, including BLM, EXO1, CtIP (CtBP interacting protein), and MRN complex (comprised of MRE11, RAD50, and NBS1) [16,17,18]. After the end resection, replication protein A (RPA) binds to the ssDNA tail, which is replaced by RAD51 to form RAD51–ssDNA nucleofilament, to search for homologous DNA sequences. Other factors, including BRCA1, BRCA2, and RAD52, are also important for the formation and stabilization of the RAD51–ssDNA nucleofilament [19,20,21]. The RAD51–ssDNA nucleofilament subsequently invades the homologous DNA sequences, and synthesizes DNA by using the homologous DNA sequences as a template. After the DNA synthesis, DNA ends are then ligated to the other side of the DSB [22]. Interestingly, RB has also been reported to promote HR and end resection by recruiting an ATPase BRG1 [23,24]. Loss of function of RB leads to impaired end resection and HR [23]. Recently, the same group further reported that E2F1 acetylation is important for the recruitment p300/CBP to promote end resection [25]. It has also been reported that loss of RB causes DNA replication fork stalling, suggesting that RB could be important for repairing DNA replication-related DSBs [26]. Loss of HR or C-NHEJ could lead to the use of the alternative and more mutagenic micro-homology mediated end joining (MMEJ) to repair DSBs [27]. Previously, MMEJ has also been shown to contribute to genome instability. In MMEJ, a short-range end resection is required to generate micro-homology regions [28]. Several important factors such as DNA Ligase III, poly-ADP ribose polymerase 1 (PARP1), and polymerase theta are required for MMEJ [29,30]. This pathway is highly mutagenic, and could contribute to chromosomal alterations, including translocations, deletions, and telomere fusions [29]. Currently, it is still debatable whether MMEJ is a backup repair pathway when HR and C-NHEJ are inhibited [31]. It has been reported that suppressing MMEJ could enhance cell death in HR deficient tumors [32]. As RB deficient cells have been reported to show impaired HR and C-NHEJ, suppressing MMEJ in RB deficient cells could be explored to develop tumor specific treatments in retinoblastoma and other RB negative cancers [9,23].

In this study, we aimed to investigate the mechanistic roles of RB in regulating the multiple DSB repair pathways. We can report that RB is required for regulating DSB repair pathway choice, through mediating CtIP dependent end resection. We propose that the limited degree of end resection inhibits both HR and C-NHEJ, and instead channels the DSB repair pathway into MMEJ. Based on these findings, the inhibition of MMEJ could be developed as a novel treatment for retinoblastoma and other RB deficient cancers.

## 2. Results

### 2.1. RB Depletion Caused Reduction in HR and C-NHEJ But Elevation in MMEJ Efficiencies

**Figure 1:**
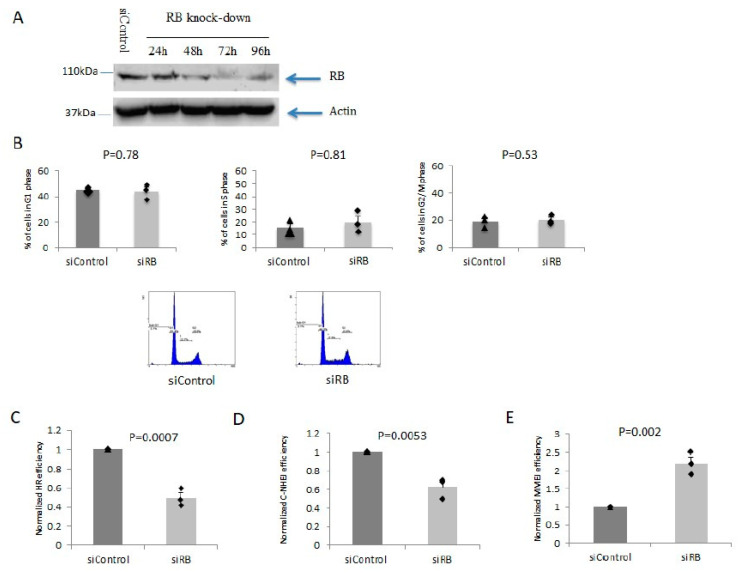
RB knocked-down cells repair double strand breaks (DSBs) mainly by the micro-homology mediated end joining (MMEJ) pathway. (**A**): RB was knocked down by siRNA treatment. (**B**): Cell cycle distribution in both RB knocked-down cells and the control treated cells. Representative cell cycle profiles are shown from one of the triplicate experiments. (**C**): Homologous recombination (HR) efficiency was measured by the DRGFP reporter assay. (**D**): C-NHEJ efficiency was measured by the EJ5GFP reporter assay. (**E**): MMEJ efficiency was measured by the EJ2GFP reporter assay. All experiments were repeated three times. Bar charts show the mean values from three experiments. Error bars show the standard error of the mean. Unpaired T-test was used for statistical analysis.

First, we established a cellular system to evaluate the roles of RB in various DSB repair pathways. We found that RB protein could be significantly depleted in U2OS cells after three days of siRNA treatment (Figure 1A). Under this RB depletion protocol, there was no significant alteration in the cell cycle distribution (Figure 1B). Furthermore, we quantified the efficiencies of HR, C-NHEJ, and MMEJ in RB knocked-down cells by separately using plasmid based assays [33]. We found that the efficiencies of HR and C-NHEJ were both decreased (Figure 1C,D). Conversely, the efficiency of MMEJ was significantly elevated (Figure 1E). Our results suggested that RB depletion led to an alteration in DSB repair efficiencies, which was independent from its cell cycle distribution regulation.

### 2.2. A Lower Degree of Resection Was Indirectly Detected in RB Knocked-Down Cells

We wanted to quantify end resection by detecting the RPA and native BrdU foci. In U2OS cells without exogenous DSB induction, RB knockdown led to less detectable RPA and native BrdU foci (Figure S1A). Cells were also studied in enhanced DSB conditions. DSBs were induced in U2OS cells by inhibiting topoisomerase I after incubating in 150nM camptothecin (CPT) for 24 h. In CPT treated RB knocked-down cells, less RPA were detected in comparison to the scrambled siRNA treated cells (Figure S1A). RPA is an ssDNA binding protein. During DSB end processing, ssDNA could be generated during the 5′ to 3′ resection of DSB ends. Alternatively, ssDNA could also be generated by unwinding DNA strands during DNA replication. We found equal RPA expression levels in RB knocked-down cells and control cells (Figure S1B). To confirm that the RPA foci could be used to represent ssDNA generated from DSB, we quantified the co-localization between RPA and γH2AX foci, which marked the positions of DSBs [34]. Our results showed extensive co-localization between RPA and γH2AX foci: 91% of γH2AX foci co-localized with RPA foci, while 88% of RPA foci contained γH2AX (Figure S1C). We showed that there were diminished levels of ssDNA in close proximity to DSBs in RB knocked-down cells, suggesting less extensive DSB end resection in these cells. The results also showed less native BrdU foci in RB-depleted cells compared with control cells. Less BrdU foci positive cells were found in RB knocked-down cells, which further confirmed less resection in RB knocked-down cells (Figure S2A). MRE11 is an important DNA break sensor, which forms the MRN complex with RAD50 and NBS1. On the other hand, it has been shown that E2F1 interacts with the N-terminal of NBS1; thus, E2F1 can be recruited to DSBs [25]. It is possible that RB has direct or indirect roles for regulating the MRN complex. In addition, the MRN complex works with phosphorylated CtIP to promote DNA end resection for both HR and MMEJ. CDK2-dependent CtIP phosphorylation could stimulate the bidirectional MRE11 nuclease activity to promote end resection [32,35]. RB knocked-down cells showed a similar amount of γH2AX foci positive cells after 150nM CPT treatment for 24 h (Figure S2B). In addition, RB knockdown did not affect MRE11 expression and foci formation after DSB induction by CPT treatment (Figure S2C), which implied that RB’s roles in regulating DNA end resection and DSB repair pathway choice are not associated with differences in DSB levels and MRE11 foci formation.

### 2.3. RB Regulated CtIP Foci Formation

**Figure 2:**
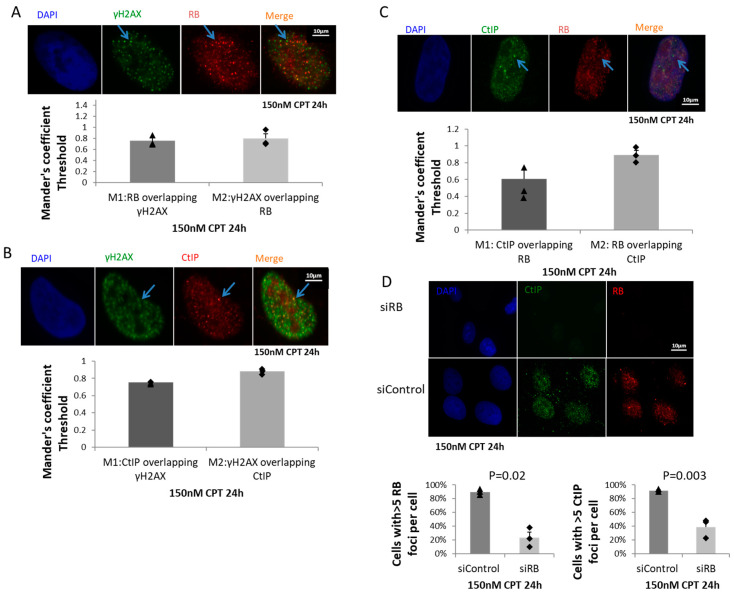
RB co-localized with CtIP at DSBs and regulated CtIP foci formation. (**A**): RB and γH2AX were co-stained by immunofluorescence after treating cells with 150 nM CPT for 24 h. Scale bar is 10 μm. Co-localization of RB and γH2AX was quantified by ImageJ software. In total, 115 cells were counted. Manders’ coefficients showed the percentages of co-localization of RB and γH2AX. Experiments were repeated three times. Bar chart shows the mean values from three experiments. Error bars show the standard error of the mean. Blue arrows indicate an example of RB and γH2AX co-localization. (**B**): CtIP and γH2AX were co-stained by immunofluorescence after treating cells with 150 nM CPT for 24 h. Scale bar is 10 μm. Co-localization of CtIP and γH2AX was quantified by ImageJ software. In total 183 cells were counted. Manders’ coefficients showed the percentages of co-localization of CtIP and γH2AX. Experiments were repeated three times. Bar chart shows the mean values from three experiments. Error bars show the standard error of the mean. Blue arrows indicate an example of CtIP and γH2AX co-localization. (**C**): RB and CtIP were co-stained by immunofluorescence after treating cells with 150 nM CPT for 24 h. Scale bar is 10 μm. Co-localization of RB and CtIP was quantified by ImageJ software. In total 102 cells were counted. Manders’ coefficients showed the percentages of co-localization of RB and CtIP. Experiments were repeated three times. Bar chart shows the mean values from three experiments. Error bars show the standard error of the mean. Blue arrows indicate an example of RB and CtIP co-localization. (**D**): RB and CtIP were co-stained in both RB knocked-down cells and control treated cells. Experiments were repeated three times. In total 102 cells in the siControl, and 138 cells in the siRB, groups were counted. Bar charts show the mean value from three experiments. Error bars show the standard error of the mean. An unpaired T-test was used for statistical analysis.

In cells treated with 150nM CPT for 24 h, we found that both RB and CtIP showed high levels of co-localization with γH2AX (Figure 2A,B). In addition, we found a high level of co-localization of RB and CtIP foci: 89% of RB foci co-localized with CtIP foci, while 61% of CtIP foci contained RB (Figure 2A). We further investigated the roles of RB in regulating CtIP foci formation. In CPT treated RB knocked-down cells, significantly less CtIP foci could be detected (Figure 2B). Our results indicated that RB is important for CtIP foci formation.

### 2.4. B Was Important for the T847 Phosphorylation on CtIP 

**Figure 3:**
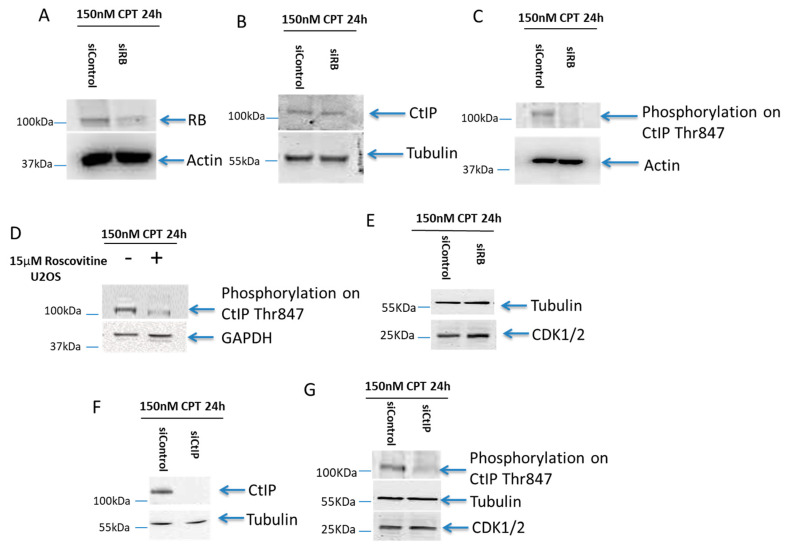
RB regulated CDK2-mediated CtIP phosphorylation on Thr847. (**A**): RB was knocked down in cells treated with 150nM CPT. Actin was used as the loading control (**B**): Equal expression levels of CtIP were found in both RB knocked-down cells and control treated cells treated with 150nM CPT for 24 h. Tubulin was used as the loading control (**C**): A lack of phosphorylation on CtIP T847 was observed in RB knocked-down cells treated with 150nM CPT for 24 h. Actin was used as the loading control (**D**): Less phosphorylation on CtIP T847 was observed in U2OS cells treated with 15 μM CDK2 inhibitor, roscovitine, and 150nM CPT for 24 h. GAPDH was used as the loading control. (**E**): Equal expression level of CDK1/2 in both RB knocked-down cells and control treated cells treated with 150nM CPT for 24 h. Tubulin was used as the loading control. (**F**): CtIP was knocked down in cells treated with 150nM CPT. Tubulin was used as the loading control. (**G**): A lack of phosphorylation on CtIP Thr847 was detected in CtIP knocked-down cells treated with 150nM CPT for 24 h. An equal expression level of CDK1/2 was detected in CtIP knocked-down cells and control treated cells. Tubulin was used as the loading control.

To further study the interaction between RB and CtIP, we also examined the CtIP expression level in RB-depleted cells. RB knockdown deficiency is shown in Figure 3A. A similar expression level of CtIP was observed in both CPT treated RB knocked-down, and scramble siRNA treated, cells (Figure 3B). To test our hypothesis of RB regulating phosphorylated CtIP mediated resection, the Thr847 phosphorylated CtIP level was observed by western blot in CPT treated RB knocked-down cells. A significantly lower level of Thr847 phosphorylated CtIP could be detected (Figure 3C). The phosphorylation of CtIP on Thr847 was suppressed in cells co-treated with 15µM roscovitine, an inhibitor of CDK2, and 150nM CPT for 24 h (Figure 3D). Our results demonstrated that RB is needed for the CDK2-mediated phosphorylation of CtIP on Thr847. However, the expression of CDK1/2 was not altered in RB knocked-down cells (Figure 3E). In addition, when CtIP was knocked down, no change in the expression of CDK1/2 was observed (Figure 3F,G). Our results indicate that RB could directly regulate the phosphorylation of CtIP on Thr847.

### 2.5. RB-Depleted Cells Were Hypersensitive to the Combined Treatment of PARP1 Inhibitor and Camptothecin

**Figure 4:**
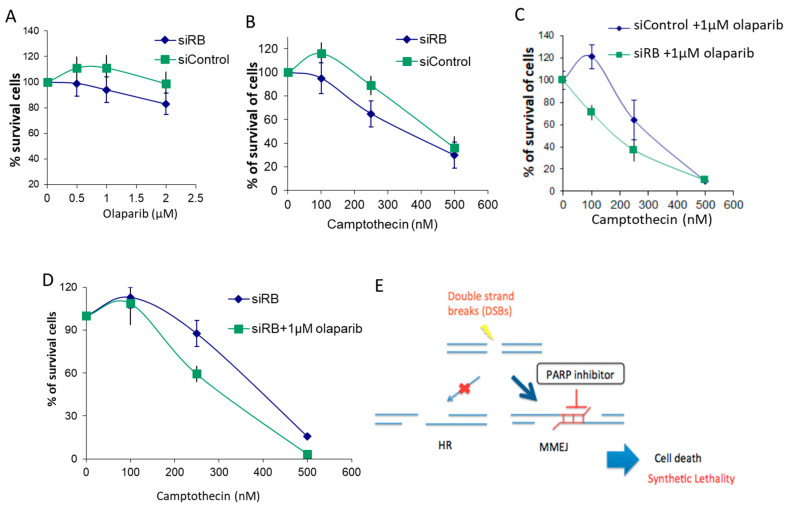
RB knocked-down cells were hypersensitive to the combined treatment of poly-ADP ribose polymerase 1 (PARP1) inhibitor and camptothecin. (**A**): Cell survival was quantified by MTT assay in RB knocked-down cells with different dosages of olaparib for 3 days. (**B**): Cell survival was quantified by MTT assay in RB knocked-down and control cells treated with different dosages of CPT for 3 days. (**C**): Cell survival was quantified by MTT assay in RB knocked-down and control cells treated with different dosages of CPT with 1 μM olaparib for 3 days. (**D**): Cell survival was quantified by MTT assay in RB knocked-down cells with different dosages of CPT, with or without 1 μM olaparib, for 3 days. Mean values from three experiments are shown. Error bars show the standard error of the mean. Experiments were repeated three times. (**E**): RB-deficient cells are HR deficient and depend on MMEJ to repair DSB-repair. After DSB induction by CPT, PARP1 inhibitor could effectively block MMEJ, and led to enhanced cell death. The light red arrow indicates the HR pathway is inhibited in RB-deficient cells. The dark blue arrow indicates the MMEJ pathway is preferred in RB-deficient cells. The light blue arrow shows the synthetic lethality in RB-deficient cells treated with PARP inhibitor.

Our results showed that RB-depleted cells repair DSBs by employing the MMEJ pathway. As RB-depleted cells showed lower HR and C-NHEJ efficiencies, while the MMEJ efficiency was elevated, we hypothesized that RB-depleted cells would be more sensitive to PARP1 inhibition, and also to CPT with PARP1 inhibitor, olaparib, co-treatment, than the single CPT treatment. We found that RB knockdown led to less cell survival in response to olaparib and to camptothecin (Figure 4A,B). This result is consistent with a previous study [23]. In addition, our results demonstrated a synthetic lethality in RB-depleted cells in response to olaparib and CPT co-treatment. After inducing DSBs with CPT, enhanced sensitivity was observed in RB knocked-down cells co-treated with CPT and olaparib, compared with the control cells (Figure 4C). Furthermore, RB knocked-down cells showed an enhanced effect in co-treatment with CPT and olaparib, compared with single treatment with CPT (Figure 4D). As resection is largely initiated in the S/G2 phase [36], PARP inhibitor could induce replication stress and accumulate cells in the S phase. Both HR and MMEJ are more active in the S phase. PARP1 has been reported to be important in resection [37]. We suggest that the synthetic lethality observed in RB-depleted cells is related to the PARP1 inhibitor interrupting the early resection event in the MMEJ pathway, so that cells have a lower DSB repair efficiency to handle the elevated level of DSBs (Figure 4E).

PARP1 inhibitor specifically blocking MMEJ may sensitize RB-deficient cells to co-treatment with PARP inhibitor and camptothecin.

## 3. Discussion

We discovered the role of RB in regulating DSB repair pathway choice through mediating CtIP dependent end resection. In RB knocked-down cells, less CtIP foci could be detected and there was a lack of phosphorylation on CtIP Thr847; a phosphorylation event important for DNA end resection. In contrast to the diminished HR and C-NHEJ levels in RB knocked-down cells, MMEJ was elevated in these cells. PARP1 is an important factor in MMEJ [30]. We reported that PARP1 inhibitor caused additional cell death in RB knocked-down cells. Our study could explain the lesser end resection in RB-depleted cells. Considering possible differences between acute RB loss and chronic RB loss, future studies are needed to confirm our findings, by using stable cell lines that do not express RB. We propose that the limited degree of end resection inhibits both HR and C-NHEJ, and instead channels the DSB repair pathway into MMEJ. Based on these findings, inhibiting MMEJ could provide a novel treatment for retinoblastoma and other RB-deficient cancers. 

**Figure 5:**
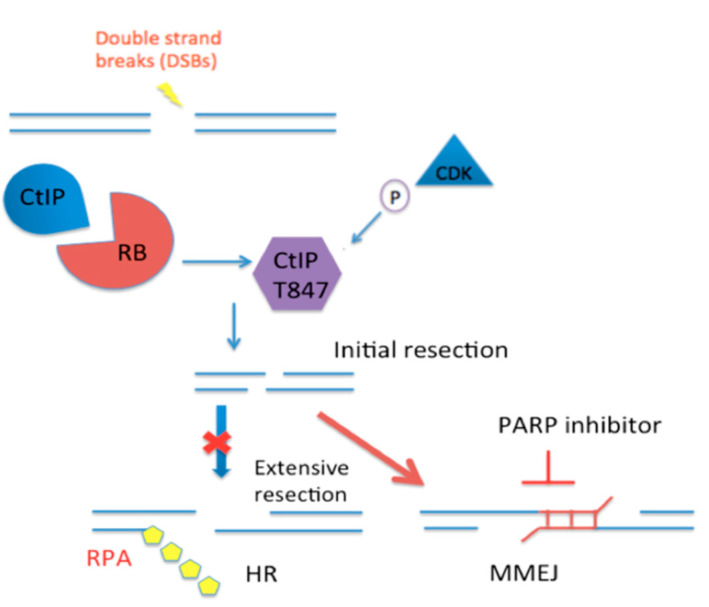
Proposed mechanism of RB in regulating CtIP mediated end resection for DSB-repair pathway choice. PARP1 inhibitor specifically blocking MMEJ may sensitize RB-deficient cells to co-treatment with PARP inhibitor and camptothecin. The red cross indicates inhibition of the extensive resection. The red arrow shows the initial resection would lead to MMEJ.

Previous studies reported RB that is important in HR and C-NHEJ [9,23]. We also found lower HR and C-NHEJ efficiencies in RB knocked-down cells. Additionally, our results suggested that genome instability is not only passively caused by the lack of HR and C-NHEJ. It could also be the consequence of the execution of an alternative DSB-repair pathway, the error-prone MMEJ pathway. MMEJ has been reported to induce chromosomal alterations, including translocations, deletions, and telomere fusions [29]. Therefore, we propose that the dysregulation of DSB-repair pathway choice could be a driving force in genome instability in RB-depleted cells. The efficiencies of these DSB-repair pathways vary along with the cell cycle progression. However, our results indicated that the dysregulation of pathway choice is independent of the cell cycle distribution. Instead, our results suggested that the DSB-repair pathway choice is regulated by the CtIP-mediated end resection. CtIP is an important protein for initiating end resection. CtIP was first identified as a RB binding protein, RBBP8 [38]. Mechanistically, we found that RB is important for the phosphorylation on T847 of CtIP, and CtIP foci formation upon camptothecin treatment. The cytotoxicity of camptothecin is S phase specific [39], when both HR and MMEJ are active. We propose a model in which RB regulates the phosphorylation on T847 of CtIP, which is a major regulator of end resection, and thus controls the DSB-repair pathway choice (Figure 5). How RB regulates CtIP phosphorylation function is currently unknown. T847 on CtIP is phosphorylated by CDK2 [40]. Moreover, CDK2 could also phosphorylate RB at the onset of the S phase [41]. In addition, CtIP has been reported to be an RB binding protein [41,42]. Future studies are needed to determine the relationships between the physical binding of RB and CtIP, and RB’s roles in activating the phosphorylation on T847 of CtIP, to regulate end resection and DSB-repair pathway choice.

Moreover, as the phosphorylation of CtIP mediated by CDK2 is a major determinant in promoting resection, RB-depleted cells employing the MMEJ pathway might not be due to impaired resection, as CtIP mediated resection is still required by MMEJ. We propose that in RB-depleted cells, CtIP is still functional, but the phosphorylation on CtIP T847 is not properly regulated, so that it channels the resected DNA towards MMEJ. It has been reported that the phosphorylation on CtIP T847 is important for resection [40]. Interestingly, another study found that in the G1 phase, CtIP T847 is important for MMEJ due to its phosphorylation by PLK3, instead of CDK2 [43]. Therefore, it is possible that in the absence of RB, the lower level of CtIP T847 phosphorylation is mediated by different kinases, which could initiate resection in G1 and S/G2, by PLK3 and CDK2, respectively, to channel the breaks towards MMEJ instead of HR. In addition, CtIP has been reported to be phosphorylated on S327 by CDK1/Aurora A, which triggers CtIP binding to PLK1, and being phosphorylated on S723 by PLK1. Phosphorylation deficiency on this PLK1 target site makes CtIP not able to promote MMEJ [44].

There are two important factors in determining the DSB-repair pathway choice. One is the initiation of DNA end resection. Our results suggested RB could regulate DSB-repair pathway choice between HR, C-NHEJ, and MMEJ through regulating end resection. In the evaluation of RB functions in resection, we found that there were less RPA foci in RB knocked-down cells after CPT treatment. RPA, bound to the ssDNA overhangs formed by resection, was able to prevent MMEJ [45,46]. Our results could not distinguish whether the reduction in RPA foci was caused by less end resection in RB knocked-down cells, or whether RB has genuine roles in recruiting RPA to ssDNA regions. Interestingly, RPA has been shown to directly bind to the amino-terminal fragment of RB [47]. Future studies are needed to understand the RPA related roles of RB. 

Another important factor in determining DSB-repair pathway choice is the speed of the repair processes. C-NHEJ repairs DSB faster, while HR is a slower procedure, because of the need of strand invasion to repair DSB. The speed of RB-depleted cells in performing MMEJ is still unknown. As HR and MMEJ are both active in the S and G2 phases, high competition could occur between these two pathways. In addition, RB may also regulate the speed of CtIP phosphorylation and resection. 

Existing treatments for retinoblastoma involve the use of chemotherapeutic agents such as carboplatin and etoposide [48]. However, these chemotherapeutic agents damage DNA, and lead to DSBs and genome instability [49]. Considering that RB-depleted cells have lower efficiencies of HR and C-NHEJ [9,23], these chemotherapeutic agents are likely to induce more unrepaired DSBs and genome instability in retinoblastoma patients. As genome instability initiates carcinogenesis, administration of these chemotherapeutic agents may lead to secondary cancers [50]. Retinoblastoma patients who are *RB1* mutation carriers have been reported to have a higher chance of secondary cancers [51]; although there is limited evidence that chemotherapeutic agents lead to secondary cancer in retinoblastoma patients. A non-DNA damaging alternative treatment is expected to cure retinoblastoma without inducing secondary cancers. Our results indicated that RB-depleted cells switch to employing MMEJ to repair DSBs. PARP1 has been reported to regulate end resection and to antagonize MMEJ [37]. Notably, our results indicated the co-treatment with CPT and PARP1 inhibitor, olaparib, further suppressed the cell proliferation in RB knocked-down cells compared to the CPT single treated cells. This result is consistent with a previous study [23]. The topoisomerase inhibitor, CPT, induced single strand breaks with blocked ends, and as the topoisomerase remains covalently bound to the break, single strand breaks could be converted into DSBs upon DNA replication. Thus, during CPT treatment, a mixture of single strand breaks and DSBs was generated [52]. Peak γH2AX accumulation occurred in S-phase cells, and was co-localized with replication foci. A previous study also demonstrated a secondary genetic lesion induced by RB loss, implying CPT response and genetic instability [53]. It has been reported that treating U2OS cells with 25nM CPT for 24 h could induce slowing and stalling of DNA replication forks, instead of generating DSBs [54]. On the other hand, treating U2OS cells with 1 μM CPT for 24 h induced massive DSBs and cell death [54]. Based on these published results, we treated cells with 150nM CPT to induce detectable levels of DSBs, without significant cell killing effects, as confirmed by the γH2AX staining and the cell proliferation assay, respectively. Under this treatment condition, we were able to study the DSB repair mechanism at elevated DSB levels in live cells. Moreover, the blocked DNA ends, due to the topoisomerase presence, also affected DSB repair. These CPT generated breaks rely greatly on CtIP for their repair, as CtIP helps with the removal of the topoisomerase, and with the resection for their repair during the S phase. Previous studies found that RB knocked-down cells showed a higher level of γH2AX, while we observed no detectable difference in RB knocked-down cells [9,23]. These studies treated cells with ionization radiation, while we induced DSBs in cells by using camptothecin. Based on these observation, we speculate that RB could still repair DSB generated by camptothecin, possibly by the elevated MMEJ pathway. On the other hand, blocked single strand breaks cause an intense activation of the PARP family (mainly PARP1) at single strand breaks [55]. Whether PARP1 inhibition interferes with the early stage of resection in RB-depleted cells will require more future studies. Furthermore, PARP inhibitor could also enhance replication stress [56]. Therefore, we propose including PARP1 inhibitors in the existing DSB inducing chemotherapies developed for treating retinoblastoma and other RB-deficient cancers.

## 4. Materials and Methods

### 4.1. Cell Culture and siRNA Transfection

Human U2OS cells were obtained from ATCC and maintained in low-glucose Dulbecco′s Modified Eagle′s Medium (DMEM) (Gibco, Walham, MA, USA) supplemented with 10% fetal bovine serum and 1% antibiotics (100 U/mL penicillin and 100 μg/mL streptomycin, Gibco). Cells were transfected with 100 nM control siRNA (siControl): 5′-UGGUUUACAUGUCGACUAA-3′ (Dharmacon, Lafayette, CO, USA), *RB1* siRNA (siRB): 5′-GAAAUGACUUCUACUCGAA-3′ (Sigma-Aldrich, St. Louis, MO, USA), or CtIP siRNA (siCtIP): 5′-GCUAAAACAGGAACGAAUC-3′ to cells with DharmaFECT1 (Dharmacon) for 72 h.

### 4.2. HR, C-NHEJ and MMEJ Assays

2.5 × 10^5^ cells were transfected with siControl and siRB for 3 days, and then were transfected with the DRGFP, EJ5GFP, and EJ2GFP to quantified HR, C-NHEJ, and MMEJ efficiencies, respectively. 2 μg DRGFP, EJ5GFP, or EJ2GFP plasmids were transfected along with pCBASceI to cells with Fugene6 (Promega, Madison, WI, USA). The ratio of Fugene6 to the transfected plasmid was 3:1. Cells transfected with 2 μg red fluorescent protein (RFP) expression plasmid pCAG-DsRed were used to normalize the transfection efficiency. Then, 72 h later, cells were collected for flow cytometry analysis. 

### 4.3. Immunofluorescence 

Cells were seeded on glass coverslips and were transfected with siControl and siRB for 2 days. Cells were then treated with 150 nM camptothecin (CPT) for 24 h. For BrdU detection, cells were treated with 10 μM BrdU for 24 h prior to the CPT treatment. After CPT treatment, cells were incubated with CSK buffer (10 mM Pipes pH6.8, 300 mM sucrose, 100 mM NaCl, 1.5 mM MgCl_2_, 0.5% Triton X) on ice for 10 min and finally washing by phosphate-buffered saline (PBS). Cells were then fixed in 3.6% para-formaldehyde in PBS for 10 min, washed in PBS, and stored at 4 °C. After permeabilization with 0.1% TritonX-100 in PBS, cells were washed in freshly made blocking buffer (0.5 g BSA, 0.15 g glycine in 50 mL PBS). Cells were then incubated with primary antibodies against RPA70 (Cell Signaling, Danvers, MA, USA, #2267, 1:200), γH2AX (Abcam, Cambridge, United Kingdom, ab26350, 1:200), BrdU antibody (Abcam, ab6326, 1:200) or MRE11 antibody (ThemoFisher, Walham, MA, USA, PA3-16527) followed by further washes with 0.1% TritonX-100 in PBS. Cells were then incubated with donkey anti-rabbit IgG AF488 (Invitrogen, Carlsbad, CA, USA, A21206, 1:800). The nuclei were mounted with DAPI (Vectorlabs, Burlingame, CA, USA) before coverslips were sealed to glass slides. RPA, γH2AX, BrdU, and MRE11 foci were visualized with a fluorescence microscope (Nikon, Tokyo, Japan) and images were analyzed by an imaging software (SPOT Imaging Solutions, Sterling Heights, MI, USA, version 5.2). Co-localization was analyzed by ImageJ software (National Institutes of Health, Bethesda, MD, USA).

### 4.4. Cell Cycle Distribution Analysis 

Cells were transfected with siRNA for 3 days. On the second day after siRNA transfection,150 nM camptothecin was added to the cells for 24 h. Cells were then harvested and fixed with 100% ethanol. The fixed cells were incubated with 100 μL PBS containing 8 μL propidium iodide and 10 μL RNase A (stock solution 10 mg/mL) at 37 °C for 30 min. Cells were then analyzed with the flow cytometer. 

### 4.5. Western Blotting

Cells were lysed in Laemmli sample buffer. After protein quantification by the BCA protein assay, protein samples were boiled with BB buffer (50 μL 1M DTT, 50 μL 0.5% Bromophenol blue (*w*/*v*) at 95 °C for 5 min. The denatured proteins were separated in sodium dodecyl sulfate–polyacrylamide gel, and then transferred to nitrocellular membranes. The membranes were probed with primary antibodies against RB (Abcam, Cambridge, United Kingdom, ab181616, 1:500), CtIP (Santa Cruz, Dallas, TX, USA, sc-271339, 1:500), phospho-Thr847 CtIP (PhosphoSolutions, Aurora, CO, USA, p1012-847, 1:500), and CDK1/2 (Santa Cruz USA, sc-53219, 1:200), followed by incubation with the appropriate secondary antibodies coupled with horseradish peroxidase. The membranes were then incubated with the enhanced chemiluminescence substrate, and images were captured.

### 4.6. MTT (Cell Viability Assay) 

Next, 1.5 × 10^6^ cells were seeded in 10 cm dishes, and treated with siControl and siRB for 48 h. Cells were then collected, and 0.1 M cells were then seeded into a 24-well plate. After 24 h, cells were treated with camptothecin, with or without olaparib, for 3 days. Cells were then incubated with 5 mg/mL MTT (3-(4,5-dimethylthiazol-2-yl)-2,5-diphenyl tetrazolium bromide) powder (Life Technologies, Carlsbad, CA, USA) in 500 μL DMEM medium at room temperature for 3 h. MTT solution were then removed and 300 μL isopropanol was added into each well. 200 μL of the mixture was transferred to a 96-well plate. Fluorescence signals were measured by a plate reader at 570 nm wavelength.

## 5. Conclusions

RB channels DSBs to be repaired mainly by HR and C-NHEJ, while it suppresses MMEJ. This DSB-repair pathway choice regulated by RB is at least partly mediated by CtIP dependent end resection. PARP1 inhibitor could suppress MMEJ, and reduce cell proliferation in RB-deficient cells. The use of PARP1 inhibitor could potentially improve treatment outcomes for RB-deficient cancers.

## Supplementary Materials

The following are available online at https://www.mdpi.com/1422-0067/21/23/9176/s1.
